# Prognostic significance of tumor-infiltrating lymphocytes in patients with operable tongue cancer

**DOI:** 10.1186/s13014-018-1099-6

**Published:** 2018-08-28

**Authors:** Wan-Yu Chen, Chen-Tu Wu, Chun-Wei Wang, Keng-Hsueh Lan, Hsiang-Kuang Liang, Bing-Shen Huang, Yih-Leong Chang, Sung-Hsin Kuo, Ann-Lii Cheng

**Affiliations:** 1 Division of Radiation Oncology, Department of Oncology, National Taiwan University Hospital, No. 7, Chung-Shan South Rd, Taipei, 100 Taiwan; 2 Graduate Institute of Clinical Medicine, National Taiwan University College of Medicine, Taipei, Taiwan; 3 Cancer Research Center, National Taiwan University College of Medicine, Taipei, Taiwan; 4 Graduate Institute of Pathology, College of Medicine, National Taiwan University, Taipei, Taiwan; 5 Department of Pathology, National Taiwan University Hospital and National Taiwan University College of Medicine, No. 7, Chung-Shan South Road, Taipei, 10002 Taiwan; 6 Department of Radiology, College of Medicine, National Taiwan University, Taipei, Taiwan; 7 Graduate Institute of Oncology, National Taiwan University College of Medicine, Taipei, Taiwan; 8 Department of Radiation Oncology, Chang Gung Memorial Hospital and Chang Gung University, Taoyuan, Taiwan; 9 Department of Internal Medicine, National Taiwan University Hospital, Taipei, Taiwan; 10 National Taiwan University Cancer Center, College of Medicine, National Taiwan University, Taipei, Taiwan

**Keywords:** Tongue cancer, Head neck cancer, Adjuvant, Tumor-infiltrating lymphocytes, Prognosis

## Abstract

**Background:**

Our aim was to investigate the prognostic significance of tumor-infiltrating lymphocytes (TILs) in operable tongue cancer patients.

**Methods:**

The presence of CD3^+^, CD4^+^, CD8^+^, and forkhead box protein P3-positive (FOXP3^+^) TILs in tumor tissues obtained from 93 patients during surgery was examined using immunohistochemistry.

**Results:**

The 3-year overall survival (OS) of patients with a low CD8/FOXP3 ratio was significantly lower than that of patients with a high CD8/FOXP3 ratio (63.8% vs. 87.3%, *p* = 0.001). Patients with high FOXP3 had a significantly lower 3-year regional recurrence-free survival (RRFS) than did patients with low FOXP3 (49.3% vs. 87.3%, univariate log rank *p* = 0.000). A low CD4/FOXP3 ratio (68.4% vs. 93.7%, univariate log rank *p* = 0.002) was significantly unfavorable prognostic factors for 3-year distant metastasis-free survival (DMFS).

**Conclusions:**

In addition to clinicopathological characteristics, TIL markers represent prognosticators for clinical outcomes.

## Background

Head and neck cancer is the sixth most common malignancy worldwide and comprises different anatomical subsites with various biological and clinical behaviors [1]. Curative treatment includes primary radiotherapy (RT) with and without different combinations of chemotherapy or curative surgery followed by adjuvant RT or concurrent chemoradiotherapy (CCRT) as clinically indicated.

An obstacle in improving the treatment outcome of head and neck cancer is immunoediting, including tumor elimination, equilibrium, and escape [2]. Tumor-infiltrating lymphocytes (TILs) are major components in the immune microenvironment. Major subtypes of TILs include CD3^+^ T cells, CD4^+^ T cells, CD8 cells, and CD4^+^CD25^+^ regulatory T cells (Treg cells; forkhead box protein P3 [FOXP3] cells). CD3 is a protein complex that functions as the coreceptor of T-cell receptor (TCR) and is required for T-cell activation; hence, CD3 is recognized as a pan-T cell marker. CD4 is a glycoprotein expressed on T cells that functions as a coreceptor of TCR and interacts with major histocompatibility complex (MHC) II on antigen-presenting cells. The interaction of CD4 with MHC II mediates a major pathway in which CD4 T cells differentiate into helper T cells and release cytokines that stimulate B cells, natural killer NK cells, and CD8 cytotoxic T cells. CD8 is expressed on cytotoxic T cells and functions as a coreceptor of TCR and binds to MHC I. CD8 cytotoxic T cells are capable of direct tumor cell killing and are vital for antitumor immunity. However, in antitumor immunity, these cells are counteracted by the immunosuppressive effect of Treg cells. Treg cells inhibit effector immune cells by direct cell–cell contact, producing immunosuppressive cytokines, such as IL-10 and TGFβ. FOXP3 is a surrogate marker for Treg and its main transcription factor [3].

Previous studies investigating the role of TILs in head and neck cancer have been limited by heterogenous anatomical sites, small sample size, and various treatment modalities. In this study, we assessed the role of TIL markers (CD3, CD4, CD8, and FOXP3) in a large cohort of patients with pure tongue squamous cell carcinoma receiving maximal curative treatment involving radical surgery and adjuvant RT or adjuvant CCRT.

## Methods

### Patients

This is a single institutional study. The study protocol was approved by the Research Ethics Committee of National Taiwan University Hospital (NTUH: 201412056RINC). The patients’ medical data were anonymized prior to access and analysis. The institutional review board waived the need for written informed consent from the study subjects because all potentially patient-identifying information was removed prior to data analysis. We included patients diagnosed with pathologically proven tongue cancer who received curative surgery and adjuvant RT with or without chemotherapy between 2004 and 2010. We excluded patients who received neoadjuvant chemotherapy or who had immune-related diseases or distant metastasis.

### Treatment

A total of 93 patients were included in this study. The patients were staged according to the AJCC sixth edition staging system. Surgical techniques included partial glossectomy and supraomohyoid neck dissection for patients with early-stage disease. Patients with locally advanced disease received nearly total or total glossectomy and modified radical neck dissection. As a cancer center, in our hospital newly diagnosed head and neck cancer patients were discussed in multidisciplinary tumor board, in terms of staging, operation technique and whether chemotherapy/targeted therapy or radiotherapy is needed or not. Surgery was performed by 4 experienced head and neck surgeons. Generally speaking, surgical tumor resection was performed with attempted 1-cm margin whenever possible. For small T/T2 tumors, tumor wide excision with adequate margin could be achieved without reconstruction. For advanced T3/4 tumors, tumor excision followed by pectoris major myocutaneous flap (PMMCF) or anterolateral thigh (ALT) flap reconstruction.

This study included patients receiving adjuvant RT for the minor pathological risk factors of a close margin (< 0.5 cm), lymphovascular invasion (LVI), perineural invasion (PNI), advanced primary tumor (T3/T4), advanced nodal status (N2/N3), or positive lymph node in neck level IV or V. Pathological N1 patients received adjuvant radiotherapy at the discretion of treating physicians. Adjuvant concurrent chemoradiation with weekly cisplatin at 40 mg/m^2^ was administered to patients with a positive margin and extracaspular extension (ECE). Patients with 2 or more minor risk factors were given chemotherapy at the discretion of treating physician. RT included 2D radiotherapy (2DRT) in 7 (7.5%) patients, 3D conformal radiotherapy (3DCRT) in 33 (35.5%), and intensity modulation radiotherapy (IMRT) in 53 (57%). The radiation field included primary tumor/neck surgical bed and bilateral prophylactic neck irradiation. The highest RT dose prescribed was 60–70 Gy to the surgical tumor bed, with 2 Gy per fraction and 5 fractions per week.

### Immunohistochemistry

Formalin-fixed, paraffin-embedded tumor specimens collected during curative surgery were used for immunostaining with primary antibodies against the following markers: CD3, pan-T cell marker (clone TR66; Enzo Life Sciences, Farmingdale, NY, USA), CD4, helper T cell marker (clone 1F6; Novocastra, Leica Microsystems, Buffalo Grove, IL, USA), CD8, cytotoxic T cell marker (clone 4B11; Novocastra, Leica Microsystems), and FOXP3, Treg cell marker (clone mAbcam 22,510; Abcam, Cambridge, UK). The representative areas containing the most densely cellular viable invasive carcinoma tissue were chosen for evaluation of TILs by experienced pathologists (YLC and CTW) blinded to the treatment and outcome. TILs that positively stained for designated markers (CD3, CD4, CD8, and FOXP3) were numerated using a 20× objective lens. The percentage of TILs positive for the following markers was calculated: CD3, CD4, CD8, FOXP3, with the ratios CD3/CD4, CD3/CD8, CD4/CD8, CD3/FOXP3, CD4/FOXP3, and CD8/FOXP3. High or low expression for each marker was defined using the median number as the cutoff point.

### Statistical analysis

The significance of the correlation of markers with clinicopathological characteristics was assessed using the Wilcoxon rank sum test. Spearman’s rank order correlation was used to determine whether a positive or negative correlation was observed among different markers. The definition for survival endpoints were as follows: Overall survival (OS): the time elapsed between operation and death from any cause; Local recurrence free survival (LRFS): the time elapsed between operation and local recurrence or death from any cause; Regional recurrence free survival (RRFS): the time elapsed between operation and regional recurrence or death from any cause; Distant metastasis free survival (DMFS): the time elapsed between operation and distant metastasis or death from any cause. Patient survival curves were generated using the Kaplan–Meier method and compared using log-rank tests. Cox’s proportional hazards regression analysis was used for multivariate analyses. Variables with a *p*-value < 0.15 were selected for subsequent multivariate analysis with entry method. Statistical analyses were performed using IBM SPSS Statistics, Version 19 (SPSS, Chicago, IL, USA). All tests were 2-sided, with significance established at *p* < 0.05.

## Results

### Clinicopathological characteristics and immunohistochemistry of TIL markers

The clinicopathological characteristics of the patients are shown in Table 1. A total of 93 patients were included in this study, with 77 (82.8%) men and 16 (17.2%) women; the median age was 49 years (range, 26–76 years). Of the 93 patients, 33 (3.5%) patients were non-smokers. The median smoking amount was 10 pack-year. Fifty-one (54.8%) patients had smoking of ≧10 pack-year. The margin status was a negative margin in 41 (44.1%) patients. Thirty-two (34.4%) patients had a close margin, and 18 (19.4%) had a positive margin. Of node-positive patients, 39 had ECE of the lymph node. Eighty (86%) patients underwent adjuvant CCRT with weekly cisplatin at 40 mg/m^2^. The median adjuvant radiation dose was 66 Gy (range, 6–70 Gy).

**Table 1:** Clinicopathologic characteristics of the patients

	Total patients
	No. (%)
Gender	
Female	16 (17.2%)
Male	77 (82.8%)
Age	
< 60 years	75 (80.6%)
≧ 60 years	18 (19.4%)
T classification	
T1/T2	52 (55.9%)
T3/T4	41 (44.1%)
N classification	
N0/N1	35 (37.6%)
N2/N3	58 (62.4%)
LVI	
No	29 (39.7%)
Yes	44 (60.3%)
PNI	
No	15 (18.7%)
Yes	65 (81.3%)
ECE	
No	36 (48%)
Yes	39 (52%)
Differentiation	
Well	17 (34.7%)
Moderate/Poor	32 (65.3%)
Margin	
Negative	41 (45.1%)
Close/Positive	50 (54.9%)
Median RT dose (cGy)	6600 (range, 600–7000)
Chemotherapy	
RT alone	13 (14%)
CCRT	80 (86%)

*Abbreviation*: *CCRT* concurrent chemoradiotherapy, *ECE* extracapsular extension, *LVI* lymphovascular invasion, *PNI* perineural invasion, *RT* radiotherapy

**Fig. 1:**
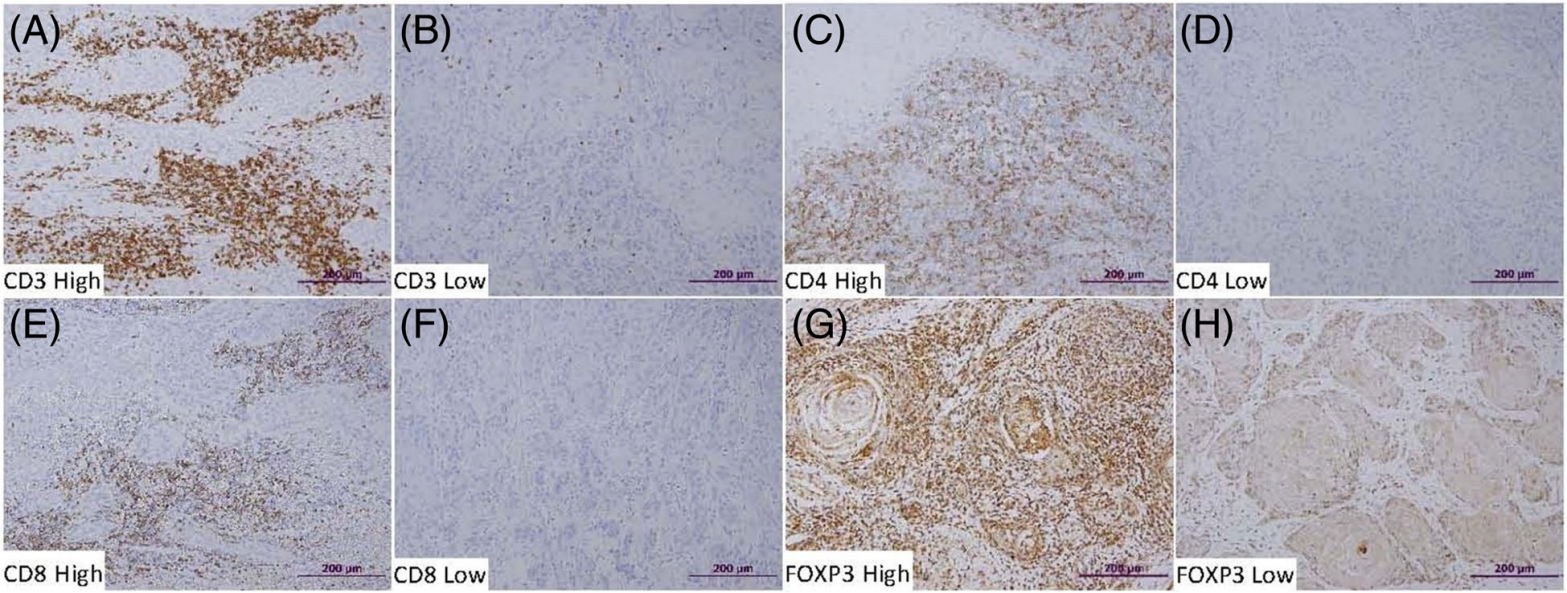
Representative specimens of immunostaining: high expression of CD3 (**a**), low expression of CD3 (**b**), high expression of CD4 (**c**), low expression of CD4 (**d**), high expression of CD8 (**e**), low expression of CD8 (**f**), high expression of FOXP3 (**g**), and low expression of FOXP3 (**h**)

The degree of intratumoral lymphocyte infiltration varied according to the marker (Fig. 1). The percentage of TILs that stained positively for CD3 (median, 70%; range 5–80; Q1-Q3, 57–70) and FOXP3 (median, 70%; range, 10–85; Q1-Q3, 70–75) was higher than that of cells positive for CD4 (median, 20%; range 1–80; Q1-Q3, 3–55), whereas the percentage for CD8 (median, 3%; range, 1–50; Q1-Q3, 1–5) was the lowest.

### Associations of TIL markers with pathological characteristics

**Fig. 2:**
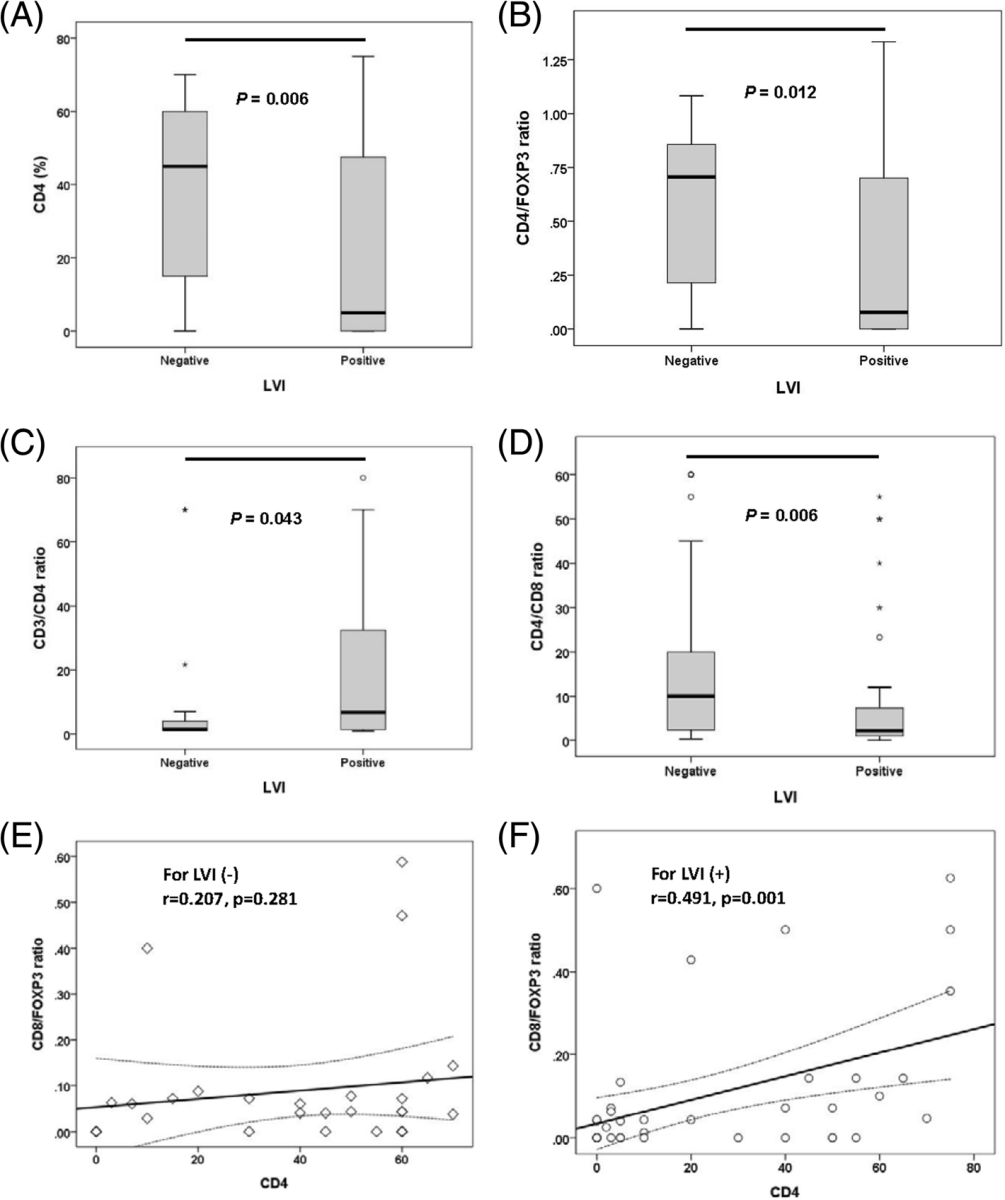
Correlation of tumor-infiltrating lymphocyte markers with pathological characteristics. The degree of correlation among CD4 (**a**), CD4/FOXP3 ratio (**b**), CD3/CD4 ratio (**c**), CD4/CD8 ratio (**d**) differs according to the pathological lymphovascular invasion (LVI) status. The correlation between CD4 and the CD8/FOXP3 ratio in LVI-negative (**e**) and LVI-positive tumors (**f**)

The percentages of TIL markers differed according to the pathological LVI status. The percentage of CD4 in LVI-negative tumors (median, 45%; range, 1–70; Q1-Q3, 13–60) was significantly higher (*p* = 0.006) than that in LVI-positive tumors (median, 5%; range, 1–75; Q1-Q3, 1–48) (Fig. 2a). The CD4/FOXP3 ratio in LVI-negative tumors (median, 0.7; range, 0–1; Q1-Q3, 0.2–0.9) was significantly higher (*p* = 0.012) than that in LVI-positive tumors (median, 0.1; range, 0.1–1.3; Q1-Q3, 0.1–0.7) (Fig. 2b). Similarly, the CD4/CD8 ratio in LVI-negative tumors (median, 10; range, 0.3–60; Q1-Q3 2–21) was significantly higher (*p* = 0.006) than that in LVI-positive tumors (median, 1.9; range, 0.1–55; Q1-Q3, 1–7.3) (Fig. 2d). By contrast, the CD3/CD4 ratio was significantly lower (*p* = 0.043) in LVI-negative tumors (median; 1.4; range, 1–70; Q1-Q3, 1.2–43) than in LVI-positive tumors (median, 7.5; range, 1–80; Q1-Q3, 1.4–32.5) (Fig. 2c).

The degree of correlation among the TIL markers also differed according to the pathological status. In LVI-positive tumors, CD4 TIL infiltration moderately correlated with the CD8/FOXP3 ratio (*r* = 0.491, *p* = 0.001) (Fig. 2f), whereas in LVI-negative tumors, the correlation of CD4 with the CD8/FOXP3 ratio did not reach statistical significance (*r* = 0.271, *p* = 0.281) (Fig. 2e).

### Prognostic role of TIL markers and clinicopathological characteristics for survival

**Fig. 3:**
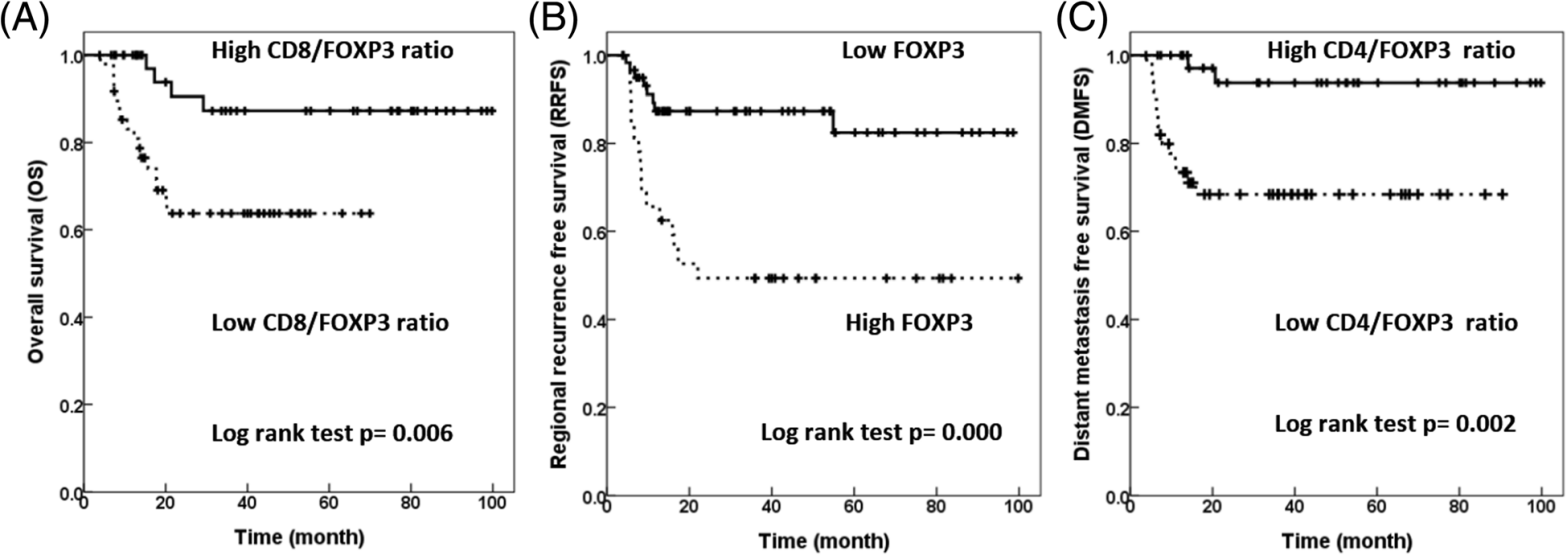
Kaplan–Meier survival curve: for (**a**) OS according to CD8/FOXP3 ratio, **b** RRFS according to FOXP3 expression, and (**c**) DMFS according to CD4/FOXP3 ratio

The median follow-up time for all patients was 31.4 months (range, 0.2–99.8 months). Table 2 summarizes the results of the univariate and multivariate analysis of the prognostic role of TIL markers and clinicopathological characteristics for OS, LRFS, RRFS, and DMFS. In univariate analysis, the 3-year OS of patients with ECE (46.2% vs. 94.4%, *p* = 0.000), N2/N3 disease (56.8% vs. 93.4%, *p* = 0.001), advanced-stage tumors (66.4% vs. 94.7%, *p* = 0.009), and a low CD8/FOXP3 ratio (63.8% vs. 87.3%, *p* = 0.001) (Fig. 3a) were significantly lower. In multivariate analysis, positive ECE (hazard ratio [HR] = 19.713, 95% CI = 2.594–149.833, *p* = 0.004) and N2/N3 (HR = 6.473, 95% CI = 1.465–28.609, *p* = 0.014) remained significant adverse prognostic factors for OS whereas a high CD8/FOXP3 ratio was a favorable prognostic factor for OS (HR = 0.015, 95% CI = 0.017–0.791, *p* = 0.028). In univariate analysis for LRFS, patients with moderate/poor differentiation (58.5% vs. 93.3%, *p* = 0.017), male sex (70.9% vs. 100%, *p* = 0.019), and close/positive margin (66.9% vs. 85.8%, *p* = 0.030) had significantly worse 3-year LRFS. Multivariate analysis showed that moderate/poor differentiation was a significant factor for LRFS (HR = 8.679, 95% CI = 1.127–66.871, *p* = 0.038).

**Table 2:** Significance of clinicopathologic parameters and TIL markers for survival

	Univariate	*p* value	Multivariate	*p* value
	3-Year survival		HR (95% CI)	
*OS*				
ECE (Yes vs. No)	46.2% vs. 94.4%	0.000	19.713 (2.594–149.833)	0.004
N classification (N2/N3 vs. N0/N1)	56.8% vs. 93.4%	0.001	6.473 (1.465–28.609)	0.014
Margin (Close/Positive vs. Negative)	60.6% vs. 80.8%	0.054		
Stage (early vs. advanced)	94.7% vs. 66.4%	0.009		
CD8/FOXP3 (High vs. Low)	87.3% vs. 63.8%	0.006	0.115 (0.017–0.791)	0.028
*LRFS*				
Differentiation (Moderate/Poor vs. Well)	58.5% vs. 93.3%	0.017	8.679 (1.127–66.871)	0.038
Gender (Male vs. Female)	70.9% vs. 100%	0.019		
Margin (Close/Positive vs. Negative)	66.9% vs. 85.8%	0.030		
ECE (Yes vs. No)	66.4% vs. 79.0%	0.095		
*RRFS*				
ECE (Yes vs. No)	57.8% vs. 86.0%	0.018	5.268 (1.503–18.466)	0.022
N classification (N2/N3 vs. N0/N1)	65.2% vs. 81.7%	0.098	16.335 (1.484–179.799)	0.024
Differentiation (Moderate/Poor vs. Well)	57.7% vs. 88.2%	0.059		
CD8 (Low vs. High)	65.9% vs. 83.2%	0.069		
FOXP3 (High vs. low)	49.3% vs. 87.3%	0.000	7.487 (1.786–31.395)	0.006
CD8/FOXP3 (High vs. Low)	84.1% vs. 61.8%	0.014		
*DMFS*				
ECE (Yes vs. No)	66.8% vs. 94.4%	0.007	3.929 (0.489–31.590)	0.042
N classification (N2/N3 vs. N0/N1)	70.3% vs. 97.1%	0.003	2.980 (0.370–24.033)	0.035
Stage (early vs. advanced)	100% vs. 72.0%	0.005		
CD3/CD4 (High vs. Low)	70.2% vs. 90.5%	0.026		
CD4/FOXP3 (High vs. Low)	93.7% vs. 68.4%	0.002	0.032 (0.003–0.384)	0.007

*Abbreviation*: *Advanced* stage 4, *CI* confidence interval, *DMFS* distant metastasis free survival, *Early stage* stage 1/2/3, *ECE* extracapsular extension, *HR* hazard ratio, *LRFS* local recurrence free survival, *OS* overall survival, *RRFS* regional recurrence free survival

We further assessed the association of the TIL markers with RRFS and DMFS of these patients. In univariate analysis for RRFS, the 3-year RRFS of patients with positive ECE and negative ECE were 57.8% and 86.0%, respectively (*p* = 0.018). Patients with N2/N3 disease (65.2% vs. 81.7%, *p* = 0.098), moderate/poor differentiation (57.7% vs. 88.2%, *p* = 0.059), low CD8 (65.9% vs. 83.2%, *p* = 0.069), high FOXP3 (49.3% vs. 87.3%, *p* = 0.000) (Fig. 3b), and a low CD8/FOXP3 ratio (61.8% vs. 84.1%, *p* = 0.014) had significantly lower 3-year RRFS. Multivariate analysis showed that in addition to the clinicopathological characteristics of ECE (HR = 5.268, 95% CI = 1.503–18.466, *p* = 0.022) and N2/N3 disease (HR = 16.335, 95% CI = 1.484–179.799, *p* = 0.024), patients with high FOXP3 still had worse RRFS (HR = 7.487, 95% CI = 1.786–31.395, *p* = 0.006). For DMFS, ECE (66.8% vs. 94.4%, *p* = 0.007), N2/N3 disease (70.3% vs. 97.1%, *p* = 0.003), advanced-stage tumors (72.0% vs. 100%, *p* = 0.005), a high CD3/CD4 ratio (70.2% vs. 90.5%, *p* = 0.026), and a low CD4/FOXP3 ratio (68.4% vs. 93.7%, *p* = 0.002) (Fig. 3c) were significantly less favorable prognostic factors for 3-year DMFS. In multivariate analysis, ECE (HR = 3.929, 95% CI = 0.489–31.590, *p* = 0.042) and N2/N3 disease (HR = 2.980, 95% CI = 0.370–24.033, *p* = 0.035) remained significant adverse factors for DMFS whereas a high CD4/FOXP3 ratio remained a favorable prognostic factor for DMFS in multivariate analysis (HR = 0.032, 95% CI = 0.003–0.384, *p* = 0.007).

## Discussion

High total TIL levels, reflecting an immune response to tumors, are associated with improved disease-specific survival (DSS) and progression-free survival (PFS) survival in human papillomavirus (HPV)-positive oropharyngeal cancer (OPSCC) [4]. Similarly, we demonstrated that abundant lymphocyte infiltrations were present in tumor microenvironments and tumor cells of our patients with tongue squamous cell carcinoma. Moreover, we further classified TIL markers into 4 groups: CD3 (pan-T cells), CD4 (CD4 helper T cells), CD8 (cytotoxic T cells), and FOXP3 (Treg cells). This study demonstrated that in LVI-negative tumors, the CD4 percentage, CD4/FOXP3 ratio, and CD4/CD8 ratio were higher, whereas the CD3/CD4 ratio was lower. Furthermore, in LVI-positive tumors, a correlation was found between CD4 and the CD8/FOXP3 ratio, whereas in LVI-negative tumors, the correlation between CD4 and CD8/FOXP3 ratio did not reach statistical significance. Similarly, Pagès et al. [5] showed that in colorectal cancer without signs of early metastatic invasion, including vascular emboli, lymphatic invasion, and PNI (collectively referred to as “VELIPI”), increased numbers of CD8^+^ T cells and high levels of messenger RNA for products of type 1 helper effector T cells and high levels of infiltrating memory CD45RO^+^ cells were observed. Our study demonstrated that in tongue squamous cell carcinoma, higher CD4 infiltration and higher CD4/CD3, CD4/CD8, and CD4/FOXP3 ratios were associated with the absence of LVI, indicating the role of CD4 TILs in the prevention of early metastatic invasion. We also found that patients with advanced-stage tumors had less CD4 and CD8, lower CD4/FOPX3 and CD8/FOXP3 ratios, and higher CD3/CD4 and CD3/CD8 ratios, which may suggest that as their tumors progressed, the tumor microenvironment shifted toward a more immunosuppressive environment.

The other key findings of this study are that patients with a low CD8/FOXP3 ratio, high FOXP3 expression, and a low CD4/FOXP3 ratio had significantly less favorable OS, RRFS, and DMFS, respectively. Previous studies have revealed that a high density of FOXP3^+^ TIL is associated with worse OS in breast cancer [6], hepatocellular carcinoma [7], and ovarian cancer [8]. In stage I lung adenocarcinoma, a high density of FOXP3^+^ TIL was also associated with shorter recurrence-free probability [9]. By contrast, a high density of FOXP3 was associated with improved survival in tumors related to the lymphoid system, such as follicular lymphoma [10] and classical Hodgkin lymphoma [11], or tumors exposed to a bacterial inflammatory environment, such as colorectal cancer [12].

In head and neck cancer, where tumors are exposed to several microbes, FOXP3 T cells were reported to be positively correlated with locoregional control [13] and OS [14]; this could be partially explained by the inhibition of harmful protumor inflammation by immunosuppressive Tregs [13, 15]. By contrast, other studies have shown that FOXP3 expression does not influence the clinical outcome of head and neck cancers [16–20]. The existence of heterogenous anatomical sites and various treatments, including surgery with adjuvant RT/CCRT or primary CCRT +/− induction chemotherapy, in previous studies may explain the differential roles of FOXP3 in the clinical outcomes of head and neck cancer. Our current study comprised a homogeneous population of patients with tongue cancer receiving curative surgery and adjuvant RT/CCRT. This finding is in accordance with that of Weed et al., who demonstrated that nuclear FOXP3 TIL was associated with tumor recurrence within 3 years in a small cohort of patients with T1/T2 tongue SCC [21]. In addition, Hanakawa et al. revealed that in tongue SCC, a high level of FOXP3 infiltration into both cancer nests and stroma correlated with worse disease-free survival [22]. The different outcomes of FOXP3 in HPV-positive oropharyngeal cancer and tongue SCC indicate the minor role of microbial-associated pathogenesis in tongue SCC. Thus, the role of FOXP3 Treg in head and neck cancer should be reinterpreted regarding the different context of subsites, respective treatment, and additional functional markers [23].

In contrast to the immunosuppressive effect of FOXP3 Treg cells, CD8 cytotoxic T cells are considered to be the major effector immune cells against tumor cells. High CD8 T-cell infiltration has been demonstrated to be associated with higher survival [14, 16, 18, 20, 24, 25] and locoregional control [17]. A low CD8/FOXP3 ratio implies a shift in the balance toward greater FOXP3 immune inhibition over the CD8 tumoricidal effect. Similarly, a low CD8/FOXP3 ratio was correlated with worse disease-free survival in tonsillar squamous cell carcinoma [20]. A low stromal CD8/CCR4 Treg ratio was an independent prognostic factor for lower survival in oral squamous cell carcinoma [24]. Our data and the findings of published studies suggest that the balance between cytotoxic T cells and Treg cells is a crucial predictive marker for the clinical outcome of head and neck cancer.

The role of CD3 and CD4 in head and neck cancer has been less documented. Balermpas et al. [16] reported that patients with high immunohistochemical CD3 expression had significantly increased OS, progress-free survival (PFS), and DMFS compared with those without. CD4 expression was not associated with clinical outcomes in most published studies [14, 16, 18, 25]. A study with a small cohort of patients with oral cavity squamous cell carcinoma showed that patients with high CD4 counts had decreased survival [26]. The CD4/CD8 ratio was associated with tumor recurrence but not OS in 52 patients with oral cancer [17].

There are several limitations in our study that should be discussed. One is its retrospective design, which introduces inherent biases. Patient source was single institution, which may result in selection bias. In addition, although two pathologist examined IHC stains independently, surgical specimen evaluation with formalin-fixed, paraffin-embedded slides could not well represent intra-tumoral heterogeneity, which is a common bias with all IHC studies.

## Conclusion

In summary, our study demonstrated that in addition to clinicopathological characteristics, TIL markers in the tumor immune microenvironment represent prognosticators for the clinical outcomes of patients with tongue cancer who received curative surgery followed by adjuvant RT with and without chemotherapy.

## Abbreviations

2DRT: 2D radiotherapy
3DCRT: 3D conformal radiotherapy
ALT: Anterolateral thigh
CCRT: Concurrent chemoradiotherapy
DMFS: Distant metastasis free survival
DSS: Disease-specific survival
ECE: Extracaspular extension
FOXP3: Forkhead box protein P3
HPV: Human papillomavirus
IMRT: Intensity modulation radiotherapy
LRFS: Local recurrence free survival
LVI: Lymphovascular invasion
MHC: Major histocompatibility complex
OPSCC: Oropharyngeal cancer
OS: Overall survival
PFS: Progress-free survival
PMMCF: Pectoris major myocutaneous flap
PNI: Perineural invasion
RRFS: Regional recurrence-free survival
RT: Radiotherapy
TCR: T-cell receptor
TILs: Tumor-infiltrating lymphocytes
Treg: Regulatory T cells
